# Hydrochemical characteristics and their controls in Jining National High-tech Zone: A land-use perspective

**DOI:** 10.1371/journal.pone.0360623

**Published:** 2026-10-14

**Authors:** Xianzhou Meng, Yifu Fu, Junye Zhang, Feng Zhang, Xiaoying Li, Hongyan An, Tianjiao Wang

**Affiliations:** 1 Shandong Provincial Lunan Geology and Exploration Institute, Shandong Provincial Bureau of Geology and Mineral Resources No. 2 Geological Brigade, Jining, Shandong, China; 2 Shandong Key Laboratory of Geothermal Clean Energy, Jining, Shandong, China; 3 Observation and Research Station of Shandong Yangzhuang Karst Ecosystem, Ministry of Natural Resources, Jining, Shandong, China; 4 Hydrogeology and Water Resources Survey Office, Langfang Integrated Natural Resources Survey Center, China Geological Survey, Langfang, Hebei, China; 5 Innovation Base for Natural Resource Monitoring Technology in the downstream area of Yongding River of the Geological Society of China, Langfang, Hebei, China; University of Sindh, PAKISTAN

## Abstract

Groundwater chemistry reflects both natural and anthropogenic processes. In rapidly urbanizing areas where agricultural and construction land coexist, differences in shallow groundwater chemistry remain poorly understood. This study compared hydrochemical characteristics between agricultural and construction land-use types in the Jining National High-tech Zone, China, using geochemical diagrams, ion ratios, mineral saturation indices, and multivariate statistics. Groundwater in the study area was generally neutral to weakly alkaline, with pH 6.78–7.51 in agricultural samples and 7.19–7.47 in construction samples. Agricultural groundwater had higher mean TDS (797.28 vs 624.00 mg/L in construction groundwater) but lower variation, suggesting spatially uniform carbonate dissolution. Conversely, construction groundwater displayed higher mean Na^+^ concentrations (59.85 mg/L) and greater hydrochemical heterogeneity. After Holm correction, pH, TDS, total hardness, and Ca^2+^ differed significantly between the two land-use types (adjusted p < 0.05), whereas the remaining parameters, including NO_3_-N, did not. The Cl^-^ coefficient of variation exceeded 100% in construction areas, and NO_3_-N variability was elevated in agricultural areas. These patterns indicate mixed influences from silicate weathering, cation exchange, and localized anthropogenic inputs. Principal component analysis yielded three components: PC1 (44.62%) represented a mineralization gradient, PC2 (17.08%) reflected pH–alkalinity balance, and PC3 (14.17%) indicated silicate weathering versus carbonate dissolution. PC1 and PC2 scores differed significantly among land-use types (p = 0.007 and 0.009, respectively), but PC3 scores did not (p = 0.826). Overall, agricultural groundwater showed carbonate-dissolution dominance with pronounced nitrate enrichment (NO_3_-N 102.7%), while construction groundwater showed silicate-influenced, Na-Cl-coupled signatures with greater heterogeneity (Cl^-^ 106.6%). These results link land-use differences to distinct shallow groundwater hydrochemical patterns, though such patterns may reflect combined influences of land use, hydrogeological setting, and aquifer lithology. The findings underscore the need for land-use-specific groundwater management in rapidly urbanizing agricultural-industrial regions and provide a baseline for future process-oriented research.

## Introduction

The chemical characteristics of groundwater are a direct reflection of the interactions between groundwater and the geological environment, providing insights into the extent of water-rock interactions as well as the impacts of human activities [1,2]. The primary ionic components of groundwater are fundamental to understanding its chemical characteristics, facilitating the identification of various factors that influence its composition [3]. As the largest available freshwater reserve globally, groundwater plays an indispensable role in supporting human livelihoods, ensuring global food security, and maintaining ecosystem integrity [4–6]. Given that more than half of the Earth's land surface is hydraulically connected to groundwater, safeguarding its quality is crucial for achieving the United Nations Sustainable Development Goals. However, rapid socioeconomic development has subjected this vital resource to increasing pressure. The combined effects of urbanization, industrialization, and agricultural expansion have led to marked alterations in regional hydrochemical conditions, resulting in the overexploitation of groundwater and the degradation of groundwater quality [7–9]. Therefore, systematic investigation of hydrochemical evolution is imperative.

The study of groundwater's hydrochemical characteristics and its spatial distribution is essential for understanding the dominant factors driving hydrochemical evolution, as well as the recharge, storage, and hydraulic relationships within water resources [10,11]. While extensive research has examined the broad hydrochemical characteristics of groundwater across various regions [12–14], comparative analyses of contrasting land-use types within rapidly urbanizing areas remain scarce. The mechanisms of groundwater ion formation are central to hydrogeological research. Interdisciplinary approaches, which integrate geochemical theories with multivariate statistical methods, have proven to be effective in elucidating the complex interactions that control groundwater composition [15–17]. Among the various anthropogenic drivers, land use represents one of the most direct and measurable influences on groundwater chemistry.

Land use is one of the most direct forms of anthropogenic influence on natural hydrogeochemical systems and has a profound effect on the chemical characteristics of groundwater [18–23]. Notably, the impacts of land use on groundwater exhibit pronounced spatiotemporal heterogeneity [24]. Although considerable progress has been made, previous studies have mainly focused on the general hydrochemical characteristics of groundwater or on a single dominant land-use type, while the comparative effects of agricultural land and construction land on shallow groundwater chemistry remain insufficiently discussed in rapidly urbanizing areas. In particular, the way in which land-use differences modify hydrochemical facies and dominant geochemical processes has not been fully clarified, and the relative weight of land use versus aquifer lithology in controlling shallow groundwater chemistry remains contested.

The Jining National High-tech Zone, established in 1992, serves as a key driver of regional economic growth and innovation. Located near the ecologically sensitive South Four Lakes and the Yellow River basin, this zone is home to a range of industrial activities, including machinery manufacturing, automotive parts production and pharmaceutical manufacturing. Despite rapid industrial expansion, agricultural land remains widely distributed throughout the region. This unique coexistence of agriculture, urbanization, and industrialization renders the area particularly suitable for investigating land-use-related hydrochemical differentiation. Although groundwater quality has received considerable attention in many rapidly developing regions, the hydrochemical evolution of shallow groundwater and its dominant controlling factors in the Jining High-tech Zone remain poorly understood. The present study focuses on shallow groundwater within the Quaternary alluvial aquifer system, which differs from deeper confined groundwater and fracture-zone groundwater in terms of recharge mechanisms, residence time, and vulnerability to surface contamination. Here, shallow groundwater is defined as groundwater stored in the intergranular pores of unconsolidated sediments, including silty clay, clay, and sand layers. Its hydrochemical characteristics reflect both natural water-rock interactions within the vadose and saturated zones and anthropogenic inputs from overlying land uses. Understanding this near-surface system is critical because shallow groundwater in the upper 5–30 m of the aquifer is more directly influenced by surface activities such as agricultural irrigation, fertilizer application, and urban runoff, whereas deeper groundwater systems are buffered from such inputs by thicker overlying strata and longer flow paths.

This study represents the first systematic comparison of shallow groundwater hydrochemistry between agricultural and construction land in a rapidly urbanizing zone. The ultimate goal is to provide robust scientific support for the sustainable management of groundwater resources in the Jining High-tech Zone. Materials and methods introduces the study area, sampling strategy, analytical methods, and data-processing approaches. Results presents the results of groundwater hydrochemical characterization under agricultural land and construction land. Discussion discusses the dominant controlling processes and the hydrochemical implications of land-use differences. Conclusion provides the main conclusions.

## Materials and methods

### Study area description

The Jining National High-tech Zone is situated in the warm temperate monsoon climate zone of southwest Shandong Province, with annual precipitation ranging from 600 to 800 mm. The region predominantly features plain depressions, with an overall elevation gradient from high in the northeast to low in the southwest. Ground elevations range from 34 to 46 m, and the average slope is approximately 0.8‰. The area's landforms are simple, consisting mainly of gently sloping flatlands, low-lying areas, and plains at the base of the mountain fronts.

The region is underlain by Quaternary alluvial and flood deposits, which were formed during the Cenozoic Era by the combined actions of the Wenhe and Sihe Rivers. The study area is located in the lower portion of the Wensi River alluvial fan, where the Quaternary sedimentary layer ranges in thickness from 90 to 120 m [25]. The sedimentary deposits exhibit a systematic thickening-to-thinning trend from northeast to southwest, reflecting a distinct depositional pattern. Hydrologically, the shallow groundwater system is recharged primarily by infiltration of atmospheric precipitation and by seepage from the Sihe River channel, the latter being the principal recharge source. Additionally, lateral groundwater flow from the northeastern region, along with recharge from agricultural irrigation, further contributes to the system. Groundwater is generally inferred to flow southwestward, broadly following the regional topographic gradient (Fig 1). In addition to its hydrogeological characteristics, the pattern of groundwater utilization and land use in the zone exerts strong control on groundwater chemistry.

**Fig 1:**
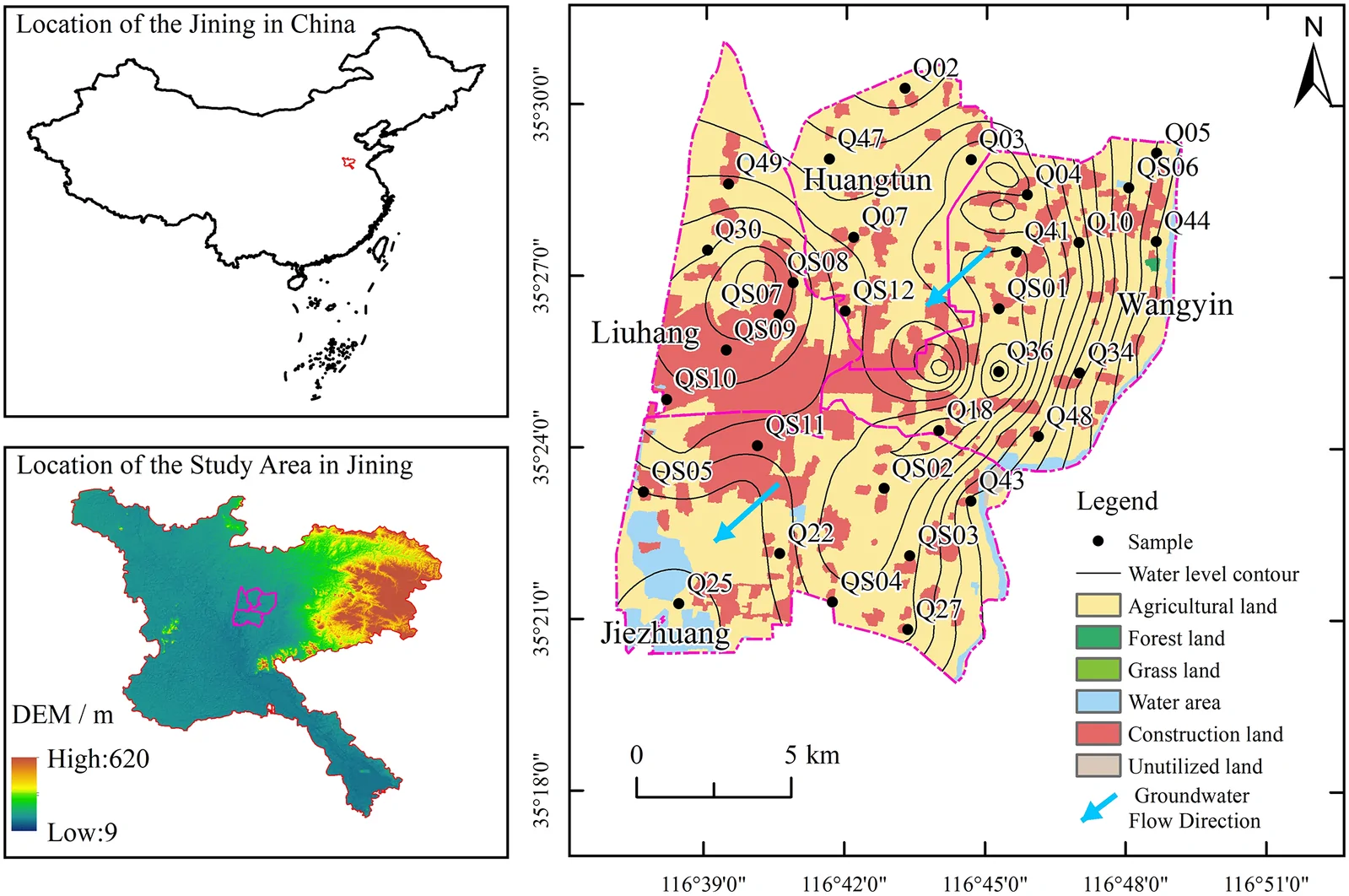
Geographical location of the study area and distribution of sampling points. Note: The coordinate reference system (CRS) is CGCS2000 and the projection center is 117°E. The administrative boundary is the China administrative division (review number GS (2024) 0650; http://bzdt.ch.mnr.gov.cn/). Land use data were obtained from the Resource and Environmental Science Data Center, Institute of Geographic Sciences and Natural Resources Research, Chinese Academy of Sciences (2020, 30 m resolution; https://www.resdc.cn; https://doi.org/10.12078/2018070201). The digital elevation model (DEM) is publicly available data from the Geospatial Data Cloud (ASTER GDEM 30M, 30 m resolution; https://www.gscloud.cn/), representing surface topography. Contour lines in the figure denote surface elevation, not groundwater hydraulic head.

Groundwater exploitation in the Jining High-tech Zone is relatively intensive, accounting for 37.31% of total water consumption in the region. Farmland and built-up areas together cover 76.23% of the zone (Fig 1). The area is primarily used for cultivating wheat and corn, covering approximately 8,967.76 hectares. Industrial activities in the zone are concentrated in manufacturing sectors, including specialized equipment manufacturing, general equipment manufacturing, automobile production, and pharmaceutical manufacturing. This landscape is characterized by the coexistence of rapid urbanization and agriculture. The land-use structure of the zone plays a pivotal role in shaping groundwater recharge, pollution migration, and chemical evolution. Understanding this structure is essential for assessing the response mechanisms of the regional groundwater system and its long-term sustainability. Given this hydrogeological and land-use context, a targeted sampling campaign was designed to capture the hydrochemical signatures associated with the dominant land-use types.

### Sampling and analysis

Field access for groundwater sampling was formally approved by the Jining High-tech Zone Management Committee. No additional permits were required as the study did not involve protected species or restricted geographic areas. Between February and March 2021, 31 shallow groundwater samples were collected from monitoring wells with depths ranging from 5 to 30 m. The sampling grid was designed to represent the primary land-use types: agricultural land (n = 18) and construction land (n = 13). Once processed, samples were sealed and transported to the Shandong Provincial Lunan Geology and Exploration Institute, Shandong Provincial Bureau of Geology and Mineral Resources No. 2 Geological Brigade Experimental Testing Center for analysis.

The bicarbonate ion concentration (HCO_3_^-^) was determined by titration, while pH was measured using a pH glass electrode. Total hardness was assessed via complexometric titration with EDTA, and total dissolved solids (TDS) were measured using a gravimetric method. Major cations (Ca^2+^, Mg^2+^, Na^+^, K^+^) and anions (F^-^, Cl^-^, SO_4_^2-^, NO_3_^-^) were analyzed using a Thermo Fisher Aquion ICS-600 Ion Chromatograph. The detection limits for all ions were below 0.01 mg/L. To ensure that the samples reflected ambient shallow groundwater, wells were purged using a centrifugal pump until stable field parameters were reached. Precise spatial coordinates were recorded with Haixingda iRTK-5 GPS equipment. Samples were filtered through 0.45 μm membranes and stored in pre-rinsed polyethylene bottles. For cation analysis, samples were acidified to a pH < 2. To ensure the reliability of the data, field blanks and blind duplicates were included in the sampling process. All samples were analyzed in triplicate, and the charge balance error (CBE) for all major ions was verified to be within ±5%. In addition to univariate and multivariate statistical analyses, geochemical modeling was employed to assess mineral equilibrium and ion exchange processes.

### Data analysis, statistics, and geochemical modeling

The hydrochemical data were processed using a multi-tool approach to differentiate between geogenic and anthropogenic controls:

IBM SPSS 25: Descriptive statistics (mean, standard deviation (SD), and coefficient of variation (CV)), Pearson correlation matrices, and hierarchical cluster analysis (HCA) were computed for the 12 hydrochemical parameters listed in Table 1. Differences between agricultural and construction samples were tested using the two-sided Mann–Whitney U test, with Holm correction applied to control the family-wise error rate across the 12 simultaneous comparisons; effect sizes are reported as Cliff's delta (δ) with bootstrap 95% confidence intervals (10,000 resamples).

**Table 1:** Descriptive statistics of groundwater parameters under agricultural and construction land of Jining High-tech Zone.

Parameter	Agricultural (n = 18)					Construction (n = 13)				
	MIN	MAX	AVG	SD	CV	MIN	MAX	AVG	SD	CV
pH	6.78	7.51	7.10	0.22	3.08	7.19	7.47	7.33	0.09	1.18
TDS	533	1284	797.28	212.33	26.63	433	1334	624.00	241.41	38.69
Ca^2+^	103	302	173.50	53.25	30.69	84	222	116.75	34.87	29.87
Mg^2+^	22.5	97.8	42.24	16.90	40.00	22	87.3	40.44	21.43	53.00
Na^+^	26.4	107	50.17	19.49	38.85	26.9	119	59.85	26.15	43.70
K^+^	0.21	2.29	0.74	0.48	65.75	0.4	2.26	0.80	0.49	60.53
Cl^-^	24.8	389	97.33	84.49	86.81	13.7	279	66.54	70.96	106.64
SO_4_^2-^	16.5	146	74.99	33.14	44.19	27.2	263	84.84	70.82	83.48
HCO_3_^-^	362	580	463.06	66.79	14.42	255	633	424.85	90.46	21.29
NO_3_-N	1.12	108	25.15	25.83	102.69	1.46	31.3	9.50	8.53	89.86
F^-^	0.36	0.88	0.61	0.15	25.32	0.33	1.27	0.65	0.29	44.93
TH	399	928	607.00	158.49	26.11	334	913	458.08	162.42	35.46

Note: “MIN,” “MAX,” “AVG,” “SD,” and “CV” denote the minimum, maximum, mean, standard deviation, and coefficient of variation, respectively. The unit of CV is %; pH is dimensionless; and all other parameters are expressed in mg/L.

Python (v3.12): Principal component analysis (PCA) was performed on z-score-standardized hydrochemical parameters using the scikit-learn library, in order to identify the dominant controls on groundwater chemistry and to reduce data dimensionality. Sampling adequacy was assessed using the Kaiser–Meyer–Olkin (KMO) measure and Bartlett's test of sphericity; the KMO value was 0.410 for the pooled dataset (agricultural, 0.389; construction, 0.403), and Bartlett's test was significant (χ^2^ = 669.52, df = 66, p < 0.001). Because the KMO value falls below the conventional 0.50 threshold, the component solution is treated as an exploratory pattern-recognition tool rather than as a definitive source-apportionment model, and component loadings are interpreted jointly with the independent geochemical evidence presented below.

PHREEQC (v3.7.3): Equilibrium modeling was conducted using PHREEQC. Major ion concentrations, pH, and temperature were considered in the modeling process. The Mineral Saturation Indices (*SI*) for calcite, dolomite, and gypsum were calculated to assess the thermodynamic tendency for mineral dissolution or precipitation [26,27]. The calculation formula for *SI* is as follows:


$$\begin{array}{c} SI=\text{lg }(\frac{\text{IAP}}{\text{K}}) \end{array}$$ (1)

where IAP is the ion activity product, and K is the solubility equilibrium constant, and ion concentration are expressed in meq·L^-1^.

The Chlor-Alkali Index (CAI) can reveal the direction and intensity of cation exchange [28]. The calculation formula for CAI is as follows:


$$\begin{array}{c} \text{CAI}-I=\frac{{\text{Cl}}^{-}-({\text{Na}}^{+}+{\text{K}}^{+})}{{\text{Cl}}^{-}} \end{array}$$ (2)

$$\begin{array}{c} \text{CAI}-\text{II\{}=\}\frac{{\text{Cl}}^{-}-({\text{Na}}^{+}+{\text{K}}^{+})}{{\text{SO}}_{\text{4}}^{\text{2}-}+{\text{HCO}}_{\text{3}}^{-}+{\text{NO}}_{\text{3}}^{-}} \end{array}$$ (3)

OriginPro 2024: This software was employed for geochemical diagnostic plotting using ion ratios and milliequivalent concentrations. The diagrams produced included Piper diagrams (for facies classification), Gibbs diagrams (for mechanism identification), and ion-relationship scatter plots (for distinguishing between weathering and evaporation processes).

### GIS and cartography

Finally, the spatial distribution of hydrochemical parameters was mapped to visualize hydrochemical evolution along the identified groundwater flow paths. ArcGIS 10.8 was used for spatial interpolation and mapping. For Fig 1, GPS coordinates (CGCS2000) and hydrochemical attribute data were integrated to generate the sampling location maps. The terrain base map was rendered from the ArcGIS online map; the digital elevation model (ASTER GDEM, 30 m resolution) was obtained from the Geospatial Data Cloud (https://www.gscloud.cn/), and the land-use data were sourced from the Resource and Environmental Science and Data Center, Institute of Geographic Sciences and Natural Resources Research, Chinese Academy of Sciences (2020, 30 m resolution; https://www.resdc.cn; https://doi.org/10.12078/2018070201). The administrative boundary was taken from the China Standard Map Service (review number GS (2024) 0650; http://bzdt.ch.mnr.gov.cn/), and the base map was used without modification. Spatial contour maps of major ions were produced using Inverse Distance Weighting (IDW) interpolation to visualize hydrochemical evolution along the groundwater flow path.

## Results

### Statistical analysis of hydrochemical parameters

As shown in Table 1, groundwater in both land-use types is generally neutral to weakly alkaline. The pH in agricultural groundwater ranged from 6.78 to 7.51 (mean 7.10, CV 3.08%), whereas that in construction groundwater ranged from 7.19 to 7.47 (mean 7.33, CV 1.18%). The lower CV in construction indicates relatively smaller spatial variability in pH, suggesting a more uniform buffering capacity across sampling sites. In terms of TDS, agricultural groundwater exhibited a higher mean TDS (797.28 mg/L) compared to construction groundwater (624.00 mg/L). However, the CV of TDS in construction groundwater (38.69%) was notably higher than that in agricultural groundwater (26.63%), indicating greater spatial heterogeneity in construction groundwater despite its lower average mineralization.

This contrasting pattern of lower mean concentrations but higher variability in construction land was observed for most hydrochemical parameters. For instance, the CV values of Mg^2+^, Na^+^, Cl^-^, and F^-^ were all higher in construction groundwater than in agricultural groundwater. Particularly noteworthy is that the CV of NO_3_-N in agricultural groundwater exceeded 100% (102.69%), and the CV of Cl^-^ in construction groundwater was also greater than 100% (106.64%).

With respect to major ion compositions, distinct patterns emerged between the two land-use types. Among alkaline earth metals, Ca^2+^ and Mg^2+^ were consistently higher in agricultural groundwater (173.50 mg/L and 42.24 mg/L, respectively) than in construction groundwater (116.75 mg/L and 40.44 mg/L), resulting in a higher mean total hardness in agricultural groundwater (607.00 mg/L vs. 458.08 mg/L). This pattern is consistent with enhanced carbonate weathering and dissolution in agricultural areas. By contrast, the mean construction of Na^+^ and K^+^ were slightly elevated in construction groundwater (59.85 mg/L and 0.80 mg/L, respectively) relative to agricultural groundwater (50.17 mg/L and 0.74 mg/L), which may reflect the combined influence of silicate weathering, cation exchange, and anthropogenic inputs associated with urban activities.

Regarding major anions, HCO_3_^-^ was the dominant anion in both land-use types, with a higher mean concentration in agricultural groundwater (463.06 mg/L, CV 14.42%) than in construction groundwater (424.85 mg/L, CV 21.29%). The relatively low CV of HCO_3_^-^ in agricultural land suggests a stable and regionally dominant control by carbonate dissolution. Conversely, SO_4_^2-^ showed a higher mean in construction groundwater (84.84 mg/L) compared to agricultural groundwater (74.99 mg/L), with substantially greater variability (CV 83.48% vs. 44.19%). Furthermore, Cl^-^ displayed a contrasting pattern: its mean concentration was higher in agricultural groundwater (97.33 mg/L) than in construction groundwater (66.54 mg/L), yet its spatial variability was markedly stronger in the latter (CV 106.64% vs. 86.81%).

Of particular concern are the nitrogen and fluoride indicators of anthropogenic influence. The mean NO_3_-N concentration in agricultural groundwater (25.15 mg/L, CV 102.69%) was substantially higher than that in construction groundwater (9.50 mg/L, CV 89.86%), with an extreme maximum value of 108 mg/L recorded at one agricultural site. This pronounced enrichment likely reflects intensive fertilizer application in cultivated areas. Meanwhile, F^-^ exhibited a slightly higher mean concentration in construction groundwater (0.65 mg/L, CV 44.93%) compared to agricultural groundwater (0.61 mg/L, CV 25.32%), suggesting more localized fluoride sources in built-up environments.

### Hydrochemical facies and spatial distribution

Beyond individual ion concentrations, the overall hydrochemical facies were classified using Piper diagrams to identify the dominant geochemical processes governing groundwater composition in each land-use type. The Piper trilinear diagram is widely used to evaluate hydrochemical evolution because it clearly shows the relative proportions and distribution patterns of major ions, helping identify the dominant end-member controls governing groundwater formation and evolution [29]. As shown in the Piper diagram (Fig 2), groundwater facies in the study area are relatively concentrated, indicating a broadly similar hydrochemical background, although clear land-use-related differences are still evident. In agricultural groundwater, the dominant water type is HCO_3_-Ca, followed by HCO_3_-Ca·Mg type and HCO_3_·Cl-Ca type. Minor occurrences of HCO_3_·Cl-Ca·Mg and Cl·HCO_3_-Ca·Mg type are also observed. Overall, these facies suggest that groundwater chemistry in agricultural areas is mainly influenced by carbonate weathering and dissolution. In construction groundwater, the dominant water type shifts to HCO_3_-Ca·Mg, followed by HCO_3_-Ca·Na type and HCO_3_-Ca type. Additional minor facies include HCO_3_·Cl-Mg·Ca type, HCO_3_·Cl-Ca·Mg type, HCO_3_-Ca·Mg·Na type, SO_4_·HCO_3_-Ca·Mg type and HCO_3_·Cl·SO_4_-Ca·Mg type. The relatively higher Na^+^ contribution in construction groundwater may reflect the combined influence of silicate weathering, cation exchange, and direct or indirect anthropogenic Na⁺ inputs. These facies differences between the two land-use types suggest distinct dominant geochemical processes, which are examined further in the Discussion.

**Fig 2:**
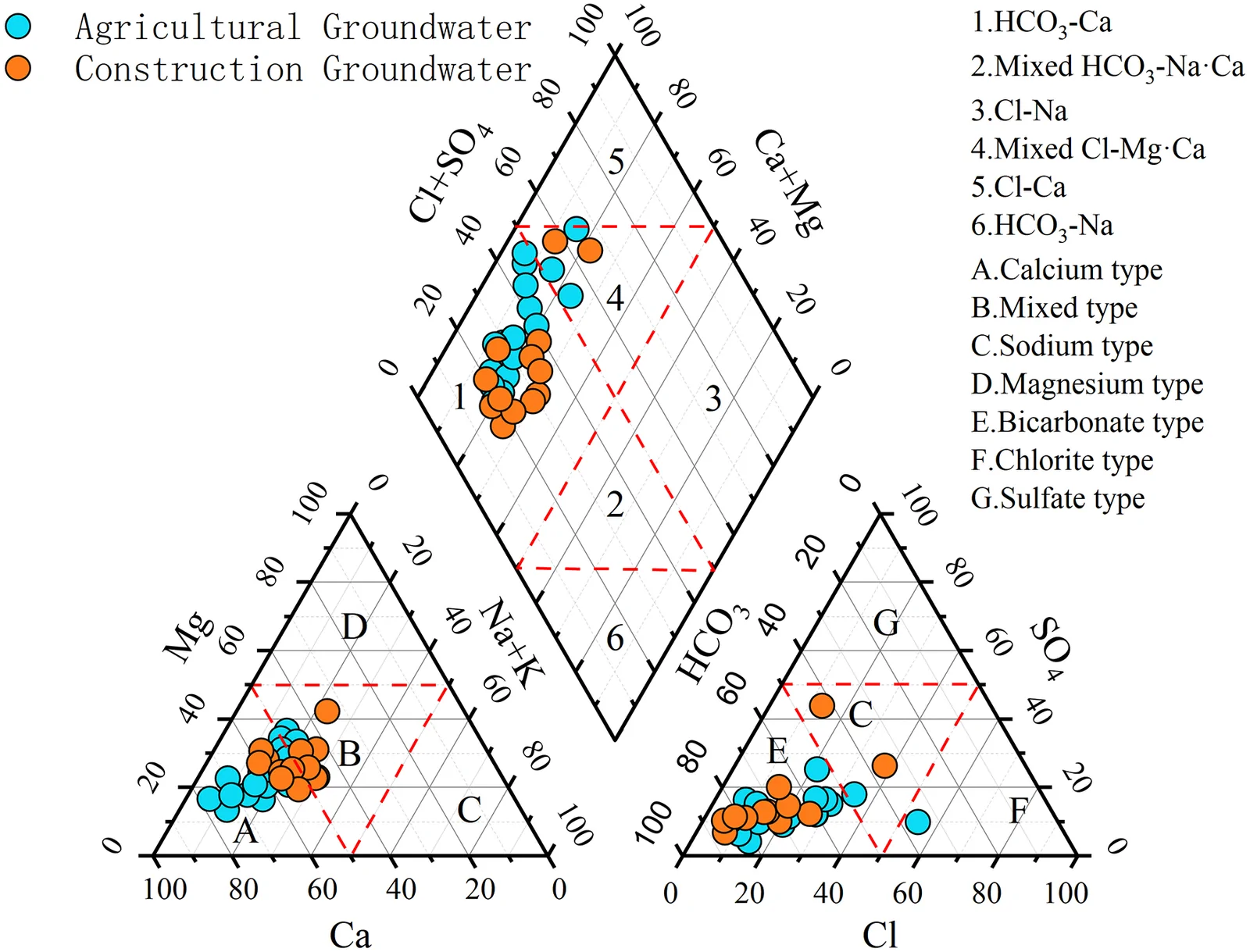
Hydrochemical piper diagram.

To further understand how hydrochemical facies evolve along the groundwater flow path, the spatial distribution of major ions was mapped (Fig 3). Overall, ion concentrations display a clear increasing trend from northeast to southwest, consistent with the regional topographic gradient and the inferred groundwater flow direction. This spatial pattern allows the identification of three distinct hydrochemical zones.

**Fig 3:**
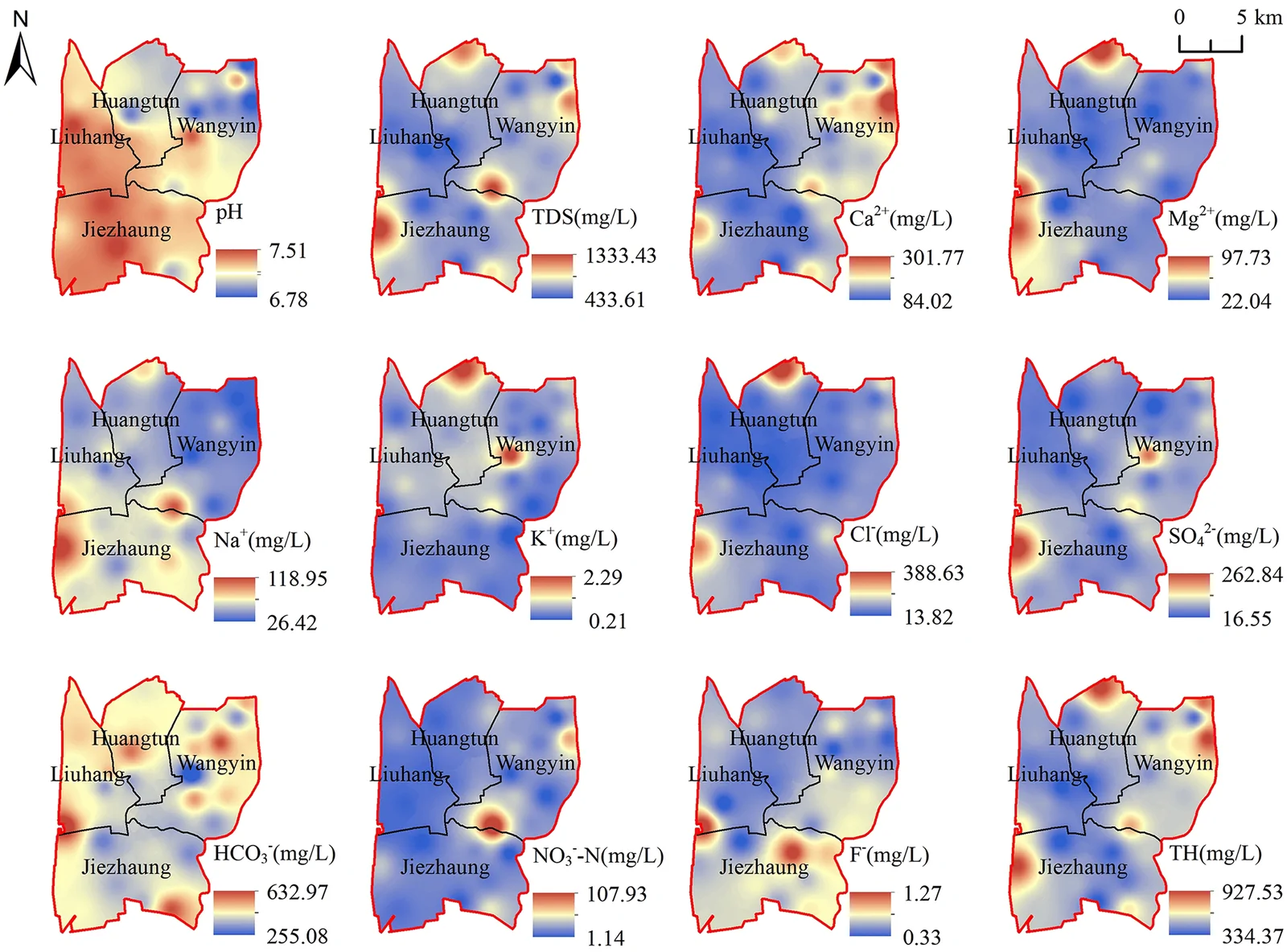
Single-factor concentration diagram. Note: The coordinate reference system (CRS) is CGCS2000 and the projection center is 117°E. The administrative boundary is the China administrative division (review number GS (2024) 0650; http://bzdt.ch.mnr.gov.cn/). The spatial distribution maps were generated by IDW interpolation based on the authors’ original sampling data provided in S1 Data.

In the upstream region, groundwater is characterized by low TDS and simple hydrochemical facies, predominantly HCO_3_-Ca and HCO_3_-Ca·Mg type, which together account for approximately 50% of the total samples in this region. These facies indicate active recharge conditions with relatively short residence times, where carbonate dissolution dominates the hydrochemical evolution. Along the flow path toward the middle region, the dominance of HCO_3_-Ca and HCO_3_-Ca·Mg type facies persists but becomes less pronounced as land-use effects become increasingly apparent. In areas dominated by construction and industrial activities, more complex facies such as Cl·HCO_3_-Ca, SO_4_·HCO_3_-Ca·Mg and HCO_3_·Cl-Ca are observed, suggesting that anthropogenic inputs introduce additional ionic sources and modify the natural geochemical evolution along the flow path.

In the southwestern discharge zone, groundwater chemistry becomes markedly more complex. HCO_3_·Cl-Ca·Mg and HCO_3_-Ca·Na types are most prevalent, accounting for approximately 60% of the samples, alongside mixed facies such as HCO_3_·Cl·SO_4_-Ca·Mg, HCO_3_·Cl-Mg·Ca, HCO_3_-Ca·Mg and HCO_3_-Ca·Mg·Na. The occurrence of these mixed facies near groundwater discharge points reflects the convergence of multiple geochemical processes: prolonged water-rock interaction along extended flow paths, evaporation-induced concentration, and enhanced mixing between groundwater and adjacent surface water bodies. Consequently, the highest ion concentrations are observed in these discharge areas, where the combined effects of extended residence time and evaporative enrichment lead to progressive mineralization of groundwater.

## Discussion

The descriptive statistics and spatial distribution patterns presented above reveal clear hydrochemical distinctions between agricultural and construction land groundwater in the Jining High-tech Zone. To elucidate the dominant processes underlying these differences, the following discussion integrates correlation analysis, hierarchical clustering, geochemical modeling, and ion ratio approaches to disentangle the relative contributions of natural geochemical controls and anthropogenic disturbances.

### Correlation and hierarchical clustering analysis

Correlation analysis is frequently employed to investigate the sources of ions in hydrochemical evolution. Similar hydrogeochemical approaches have been successfully applied to water source identification in complex mining-affected aquifers, where multivariate statistical methods effectively distinguish natural water-rock interactions from anthropogenic disturbances [1]. Components originating from the same source exhibit strong correlations, whereas those from different sources display weak correlations [30]. As shown in Fig 4 (a, b), TDS is positively correlated with total hardness (TH) and the concentrations of Ca^2+^, SO_4_^2-^, and NO_3_^-^N in groundwater (p < 0.05), indicating that the spatial–hydrogeochemical variability of TDS is largely governed by these major ions. Among them, total hardness shows the strongest positive relationship with TDS (Pearson r = 0.93; p < 0.001), followed by Ca^2+^(Pearson r = 0.82; p < 0.001), which is consistent with an important contribution of carbonate dissolution to solute accumulation along the inferred flow path. This coupling between carbonate dissolution and overall mineralization is consistent with observations in other alluvial aquifers, in which carbonate weathering sets the baseline solute load while land use modulates its magnitude [12–14].

**Fig 4:**
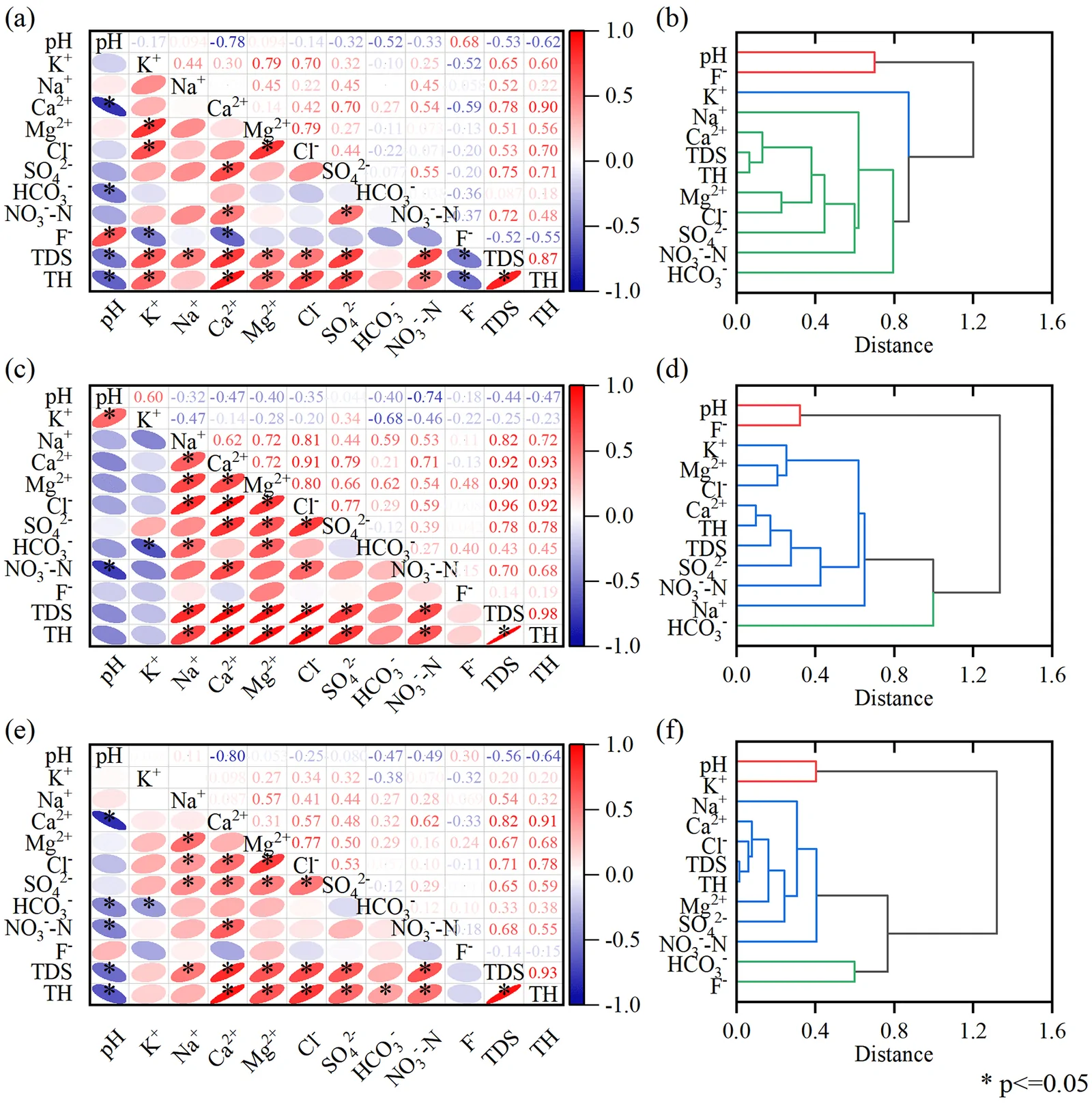
Correlation analysis group diagram. Note: K ⁺ , Na ⁺ , Ca^2+^, Mg^2+^, Cl^-^, SO_4_^2-^, HCO_3_^-^ , NO_3_^-^-N, and TDS all represent their respective concentrations, an asterisk (*) indicates that the correlation coefficient is significant at the 0.05 level, each ellipse represents the magnitude of a correlation coefficient: the flatter the ellipse, the stronger the correlation, and vice versa. A leftward tilt indicates a positive correlation, while a rightward tilt indicates a negative correlation.

The heatmaps in Fig 4 (c, d) further reveal that K^+^ and Cl^-^ cluster closely in agricultural groundwater, implying a shared or strongly coupled input. In the Jining High-tech Zone, such coupling may be associated with agricultural fertilization and irrigation return flow, both of which can facilitate the concurrent transfer of K^+^ and Cl^-^ into shallow groundwater. In contrast, construction groundwater in Fig 4 (e, f) displays a pronounced Na^+^ and Cl^-^ association, a pattern commonly linked to anthropogenic solute loading during urbanization. Leakage of domestic sewage, losses from municipal pipe networks, and inputs associated with urban construction materials may all introduce chloride accompanied by sodium, thereby enhancing NaCl-type signatures in groundwater. These statistical associations provide preliminary evidence for distinct ion sources under the two land-use types. The contrast between the K^+^ and Cl^-^ association under agriculture and the Na^+^ and Cl^-^ association under construction is notable: both pairings are widely reported as anthropogenic fingerprints, yet the two land-use types express them differently, which supports the view that land use reconfigures ion associations rather than simply adding a uniform anthropogenic signal [18–23]. The following geochemical analysis aims to identify the specific natural and anthropogenic processes responsible for these observed patterns.

To synthesize the foregoing natural and anthropogenic controls, PCA was applied to the hydrochemical dataset. The first three principal components had eigenvalues of 5.354, 2.049, and 1.701, and together explained 75.87% of the total variance (44.62%, 17.08%, and 14.17%, respectively; Table 2). PC1 representing a mineralization gradient, with strong positive loadings on TDS (0.98), TH (0.98), Ca^2+^ (0.86), Cl^-^ (0.77), Mg^2+^ (0.70), and SO_4_^2-^(0.66), and a negative loading from pH (−0.60). PC2 capturing a pH-alkalinity balance, with positive loadings from pH(0.71), Mg^2+^(0.55), and Na^+^(0.48) opposed by Ca^2+^(−0.42) and HCO_3_^-^(−0.37). PC3 distinguished silicate weathering from carbonate dissolution signatures, with positive loadings from HCO_3_^-^(0.71) and F^-^ (0.65) and a negative loading from K^+^ (−0.72). Component loadings for the pooled dataset are listed in Table 3.

**Table 2:** Eigenvalues, explained variance, and sampling adequacy of the principal component analysis.

Index	Total (n = 31)	Agricultural (n = 18)	Construction (n = 13)
Eigenvalue of PC1	5.354	5.675	6.919
Eigenvalue of PC2	2.049	2.393	2.277
Eigenvalue of PC3	1.701	1.447	1.230
Variance explained by PC1 (%)	44.62	47.29	57.66
Variance explained by PC2 (%)	17.08	19.94	18.97
Variance explained by PC3 (%)	14.17	12.06	10.25
Cumulative variance explained (%)	75.87	79.28	86.88
KMO measure of sampling adequacy	0.410	0.389	0.403

Note: Data provided in S1 and S2 Data.

**Table 3:** Component loadings of the pooled dataset (n = 31) from correlation-matrix PCA.

Parameter	PC1	PC2	PC3
pH	−0.598	0.712	0.001
TDS	0.979	0.002	0.039
Total hardness	0.975	−0.082	0.015
Ca^2+^	0.864	−0.420	−0.129
Mg^2+^	0.701	0.549	0.263
Na^+^	0.485	0.478	0.352
K^+^	0.250	0.361	−0.719
Cl^-^	0.773	0.371	−0.096
SO_4_^2-^	0.660	0.415	−0.188
HCO_3_−	0.346	−0.366	0.710
NO_3_-N	0.607	−0.339	−0.076
F−	−0.181	0.374	0.645

Note: Data provided in S1 and S2 Data.

Scores on PC1 were significantly higher for agricultural than for construction samples (mean +0.68 vs. −0.94; Mann–Whitney U test p = 0.007; Cliff's δ = 0.58), and scores on PC2 were significantly lower (p = 0.009; δ = −0.56). These two contrasts constitute the clearest statistical expression of land-use control in the dataset. PC3 scores, by contrast, did not differ between land-use types (p = 0.826; δ = 0.05). This asymmetry is informative: land use mainly shifts the position of a sample along the mineralization and pH–alkalinity axes but does not alter the silicate-versus-carbonate balance, which is governed by aquifer mineralogy rather than by surface land use.

The separation between agricultural and construction samples is therefore partial rather than complete (Table 2). Substantial overlap remains along PC2 and PC3, indicating that natural hydrogeological factors, particularly lithology, groundwater flow path length, and aquifer heterogeneity, exert controls of comparable importance to land use. It should also be emphasized that the KMO value of 0.410 lies below the conventional 0.50 threshold, and that the ratio of samples to variables is low (n/p = 2.58 for the pooled dataset and only 1.08 for the construction group). The apparent increase in the variance explained by PC1 from the agricultural (47.29%) to the construction (57.66%) subset is therefore partly a mathematical consequence of the smaller sample size and should not be read as evidence of stronger land-use control. Accordingly, the loadings reported in Table 3 are based on the pooled dataset, which is the only subset with an acceptable sample-to-variable ratio. The component solution is thus best regarded as an exploratory summary of covariance structure rather than as a definitive source apportionment, and it is interpreted here only in combination with the independent geochemical evidence (ion ratios, saturation indices, and CAI) presented above. This inherent difficulty in fully partitioning land-use signals from natural hydrogeological controls reflects a broader methodological challenge in complex Earth surface systems where natural variability and anthropogenic forcings are tightly coupled; integrated geochemical approaches must explicitly account for both sets of controls in order to avoid oversimplified source attribution [31]. The PCA results accordingly support interpreting groundwater chemistry in the Jining High-tech Zone within a coupled natural-anthropogenic framework rather than attributing it exclusively to land use.

### Analysis of influencing factors of each ion

#### Identification of control factors.

The chemical composition of groundwater is primarily shaped by atmospheric rainfall, rock weathering, evaporation concentration, and mixing. The Gibbs diagram serves as a valuable tool for inferring the hydrogeological processes governing natural water [32]. As shown in Fig 5, most of the groundwater samples fall within the “rock weathering control zone,” indicating that natural water-rock interactions are the primary determinant of water chemistry in this region. However, some agricultural groundwater samples also show a relative shift toward evaporation enrichment, as reflected by elevated Na^+^/(Na^+^ + Ca^2+^) and Cl^-^/(Cl^-^ + HCO_3_^-^) ratios. This pattern may be related to long-term agricultural practices involving irrigation and fertilization. Irrigation return flow and solute accumulation during infiltration may increase the relative proportions of Na^+^ and Cl^-^ in groundwater. This behavior is consistent with the transitional character widely reported for shallow alluvial aquifers, in which carbonate weathering governs the baseline composition while evaporation and anthropogenic inputs modulate it at the margins [20,21]. In contrast, construction groundwater samples are more concentrated within the core of the rock weathering control zone, but their ion ratios are generally lower and more tightly clustered compared to those from agricultural groundwater. The lower ion ratios suggest that the influence of evaporation concentration on groundwater is weaker in urban areas, likely due to reduced direct evaporation from urban surfaces. While these samples still fall within the rock weathering zone, their chemical composition may also be modified by external inputs, resulting in a hydrochemical pattern distinct from that of agricultural groundwater.

**Fig 5:**
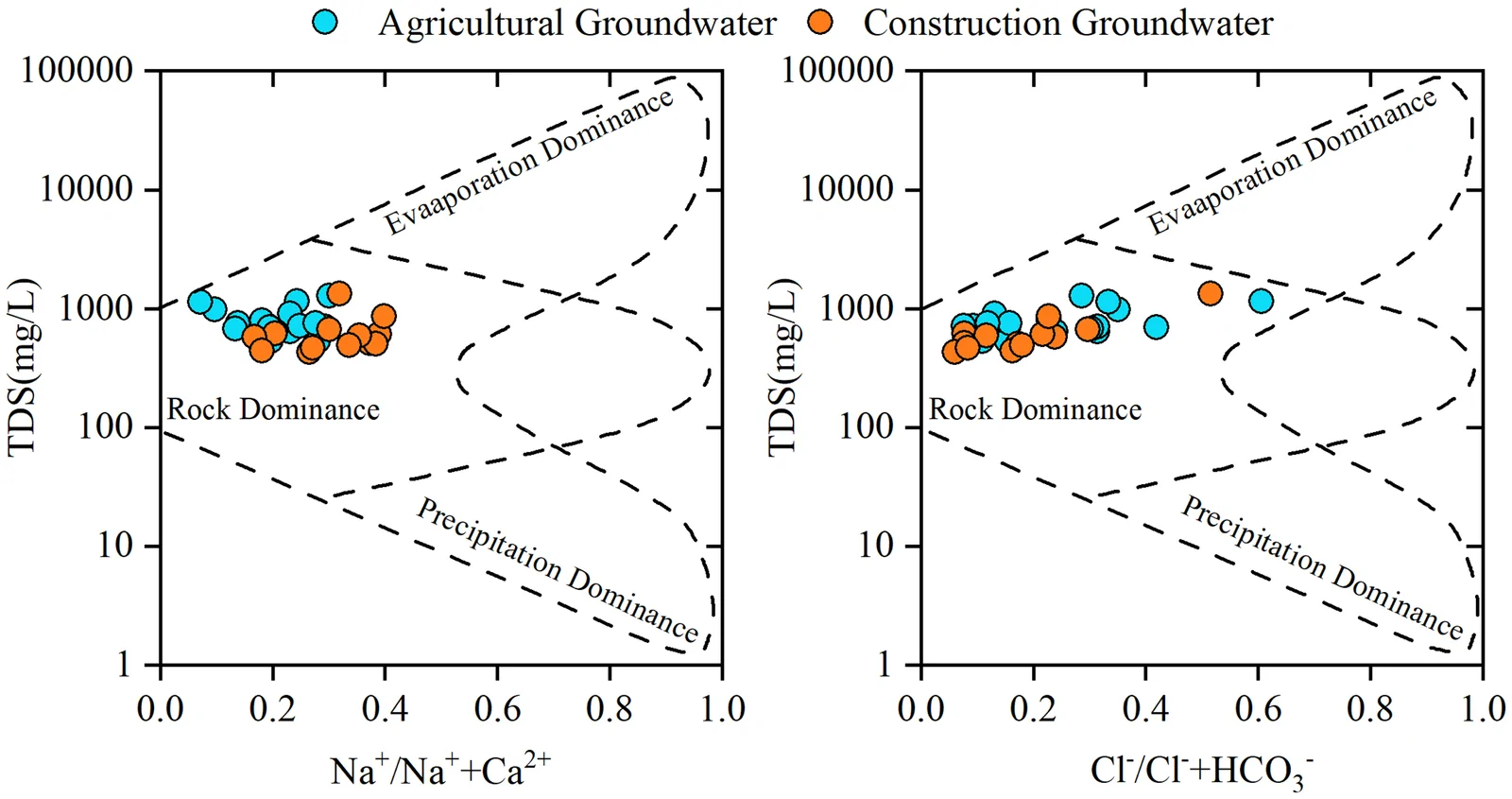
Gibbs diagram of water chemistry.

To further distinguish the relative contributions of carbonate versus silicate weathering, the relationship between Ca^2+^/Na^+^ and HCO_3_^-^/Na^+^, as well as Ca^2+^/Na^+^ and Mg^2+^/Na^+^, can help identify the impact of different rock weathering leaching on water solutes [33]. Fig 6 further highlights the variations in groundwater solute contributions resulting from distinct rock weathering processes. The groundwater samples in the study area are predominantly distributed between the silicate rock weathering and carbonate rock weathering control zones, indicating that the hydrochemical characteristics of the region are influenced by the dissolution of both types of rocks. Agricultural groundwater samples are closer to the carbonate rock end-member, with higher Ca^2+^/Na^+^ ratios than those from construction areas, indicating more pronounced enrichment in Ca^2+^, Mg^2+^, and HCO_3_^-^. This enhanced carbonate weathering signal in agricultural groundwater is largely attributed to the geochemical environment of the soil, which is influenced by agricultural irrigation processes and extended water-rock contact. Slukovskii et al. similarly noted that even pristine arctic lake systems are highly responsive to external solute inputs, underscoring the sensitivity of weakly buffered systems to land-use forcing [7]. In contrast, groundwater samples from construction areas show a shift toward the silicate rock weathering zone, as reflected in their leftward and downward movement on the diagram. This shift is likely attributable to a dilution effect or to cation exchange associated with anthropogenic activities, which shifts the ion balance toward silicate weathering characteristics.

**Fig 6:**
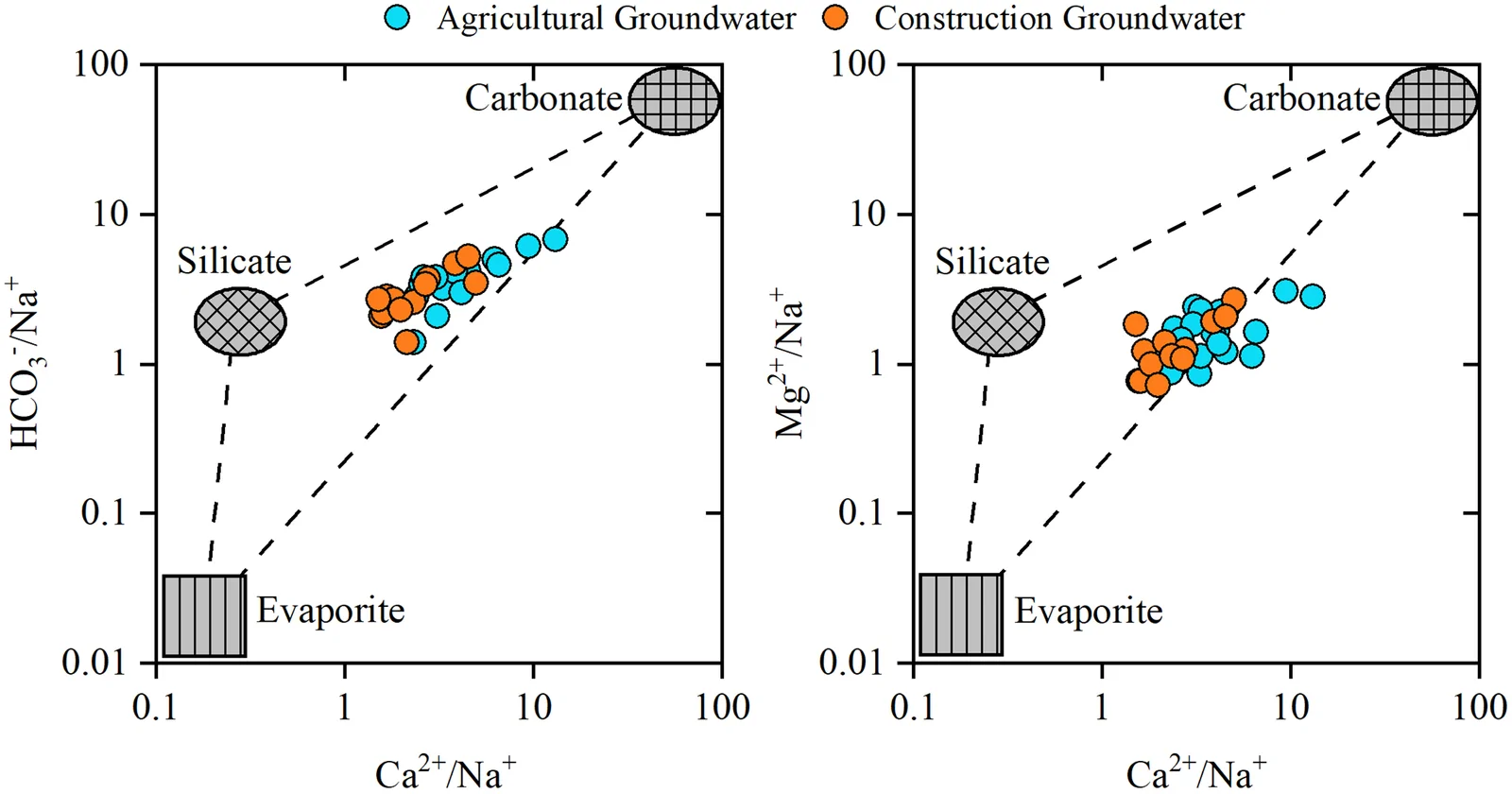
Relationship between Ca^2+^/Na^+^ and Mg^2+^/Na^+^ and Ca^2+^/Na^+^ and HCO_3_^-^/Na^+^.

#### Ion ratio analysis.

Beyond weathering type identification, specific ion ratios were examined to constrain the exact sources of major cations and anions. Na^+^+K^+^ mainly originates from the weathering and dissolution of silicate minerals and evaporite rocks. Cl^-^ is relatively stable in groundwater, primarily derived from the weathering and dissolution of evaporite rocks. The ratio of *ρ*(Na^+^+K^+^) to *ρ*(Cl^-^) helps identify the sources of Na^+^ and K^+^ in the water. A ratio of 1 indicates halite dissolution, whereas ratios >1 suggest additional Na⁺ from silicate weathering. If the ratio is less than 1, it suggests an additional Cl^-^ source, often associated with human activities [34]. As shown in Fig 7a, most construction groundwater samples exhibit a ratio greater than 1, implying that the sources of Na^+^ are not limited to halite dissolution, but also include substantial contributions from silicate rock weathering. In contrast, many agricultural groundwater samples tend to show ratios below 1, suggesting the presence of additional chloride sources. This pattern may be related to agricultural activities, including fertilizer application and irrigation-related solute transport.

**Fig 7:**
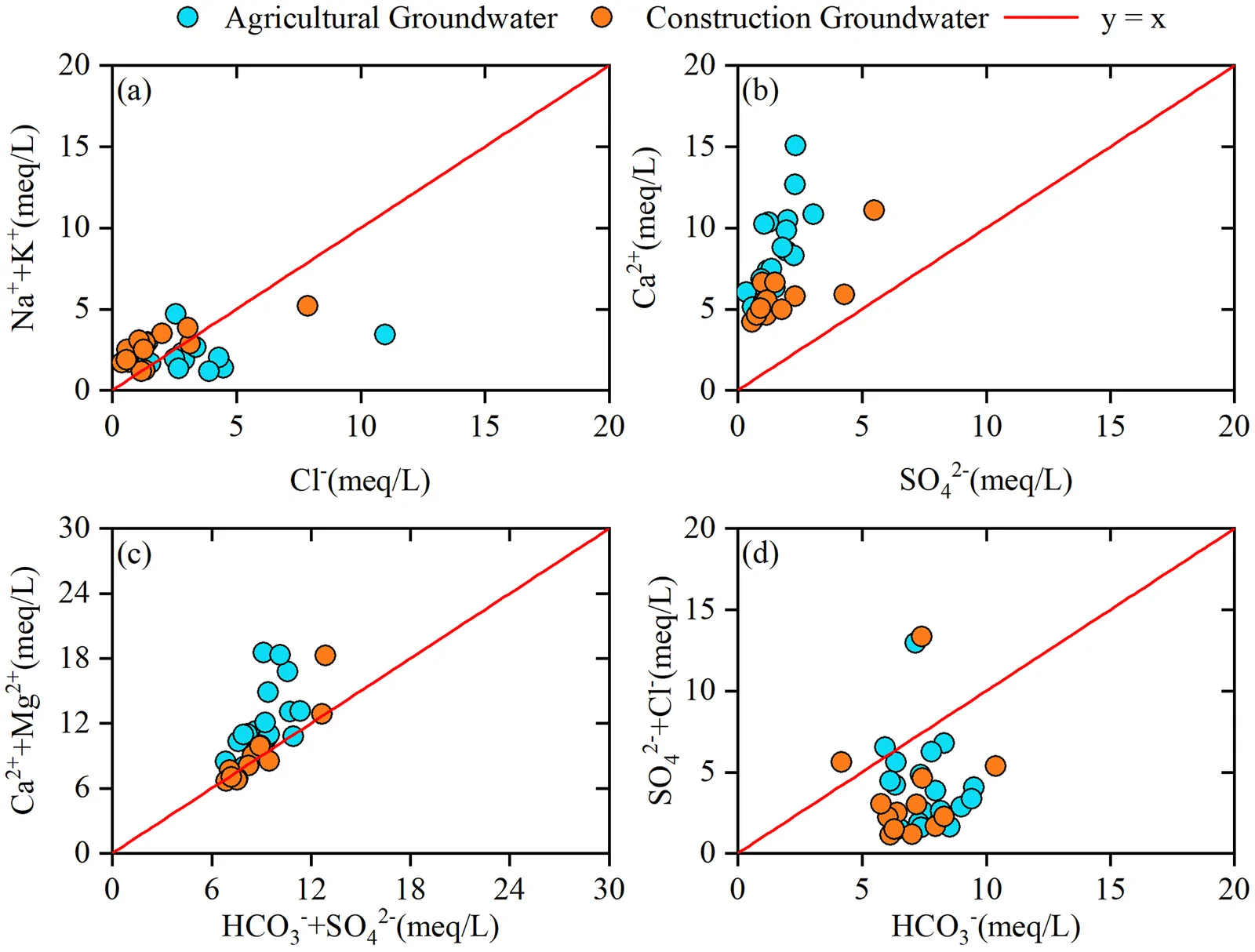
Water ratio relationship of ions.

The ratio of *ρ*(Ca^2+^) and *ρ*(SO_4_^2-^) was used to determine whether gypsum dissolution occurred [35]. Fig 7b illustrates that the majority of sample points in the study area lie above the 1:1 line, suggesting that gypsum dissolution may represent an important background source affecting groundwater chemistry.

The ions SO_4_^2-^, HCO_3_^-^, Mg^2+^ and Ca^2+^ typically originate from the weathering and dissolution processes of evaporated salt rocks, siliceous rock salts, and carbonate rocks. The ratio of *ρ*(Ca^2+^ + Mg^2+^) and *ρ*(HCO_3_^-^ + SO_4_^2-^) helps identify the sources of Ca^2+^ and Mg^2+^ in groundwater. A ratio >1 indicates that these ions mainly derive from carbonate dissolution. If the ratio is less than 1, it suggests that the weathering of silicate rocks is the dominant process [36]. As shown in Fig 7c, most agricultural groundwater samples exhibit ratios greater than 1, signifying that Ca^2+^ and Mg^2+^ primarily originate from the dissolution of carbonate rocks. In contrast, construction groundwater samples tend to fall closer to 1, suggesting that both carbonate and silicate rock weathering influence the Ca^2+^ and Mg^2+^ concentrations in these samples.

The ratio of *ρ*(Cl^-^ + SO_4_^2-^) and *ρ*(HCO_3_^-^) can help identify the sources of Cl^-^, SO_4_^2-^, HCO_3_^-^. When this ratio is less than 1, it suggests that the primary source of Cl^-^, SO_4_^2-^, HCO_3_^-^ is the dissolution of carbonate rocks. When the ratio is greater than 1, it indicates a substantial contribution from the weathering of silicate rocks to the anion composition [37]. Fig 7d reveals that approximately 87% of the groundwater samples exhibit ratios below 1, indicating that HCO_3_^-^ is predominantly sourced from the dissolution of carbonate rocks.

#### Mineral saturation analysis.

While ion ratios identify the sources of dissolved constituents, mineral saturation indices reveal the thermodynamic state of the groundwater system and its potential for further mineral dissolution or precipitation. The mineral saturation index (*SI*) is calculated using the PHREEQC software to identify the reaction state between minerals and groundwater, revealing the primary factors influencing the chemical evolution of groundwater [26,27]. Fig 8 reveals marked differences in *SI* values for groundwater from agricultural and construction areas, highlighting contrasts in water-mineral interaction. In construction groundwater, the samples are concentrated in the lower TDS range with relatively low *SI* values. This suggests a shorter residence time or closer proximity to recharge sources, where groundwater has had limited contact with aquifer minerals. The relatively low *SI* values in construction groundwater are therefore consistent with faster recharge and reduced residence time in urbanized settings. On the other hand, agricultural groundwater samples exhibit a broader range of TDS values, extending up to 1300 mg/L, along with higher *SI* values, particularly for carbonate minerals. This positive correlation between *SI* and TDS in agricultural areas indicates that processes like evaporation concentration and agricultural inputs play a major role in increasing ion concentrations. These elevated concentrations maintain a supersaturated state for carbonate minerals, contributing to the higher *SI* values seen in agricultural areas. Fig 8 (a, b) show that the *SI* values for agricultural groundwater are consistently greater than 0, suggesting supersaturation with respect to minerals, especially carbonates. This pattern may be related to stronger solute accumulation and longer water-rock interaction under agricultural-land conditions, with irrigation-related concentration effects possibly contributing. In contrast, construction groundwater has *SI* values that approach 0, indicating saturation or near equilibrium with respect to these minerals, which reflects limited mineral interaction owing to rapid infiltration in urban areas. Impervious urban surfaces accelerate groundwater movement and shorten the time available for minerals to dissolve or precipitate. In Fig 8 (c, d), gypsum and rock salt have *SI* values below 0, indicating that groundwater is unsaturated with respect to both minerals. This unsaturation suggests that the groundwater has not reached dissolution equilibrium with gypsum and rock salt, which may be due to the short residence time of shallow groundwater and the limited availability of these minerals in the aquifer matrix or relatively lower ion concentrations in the groundwater, preventing saturation. Representative input parameters and PHREEQC-derived saturation indices for selected minerals are provided in S1 Data.

**Fig 8:**
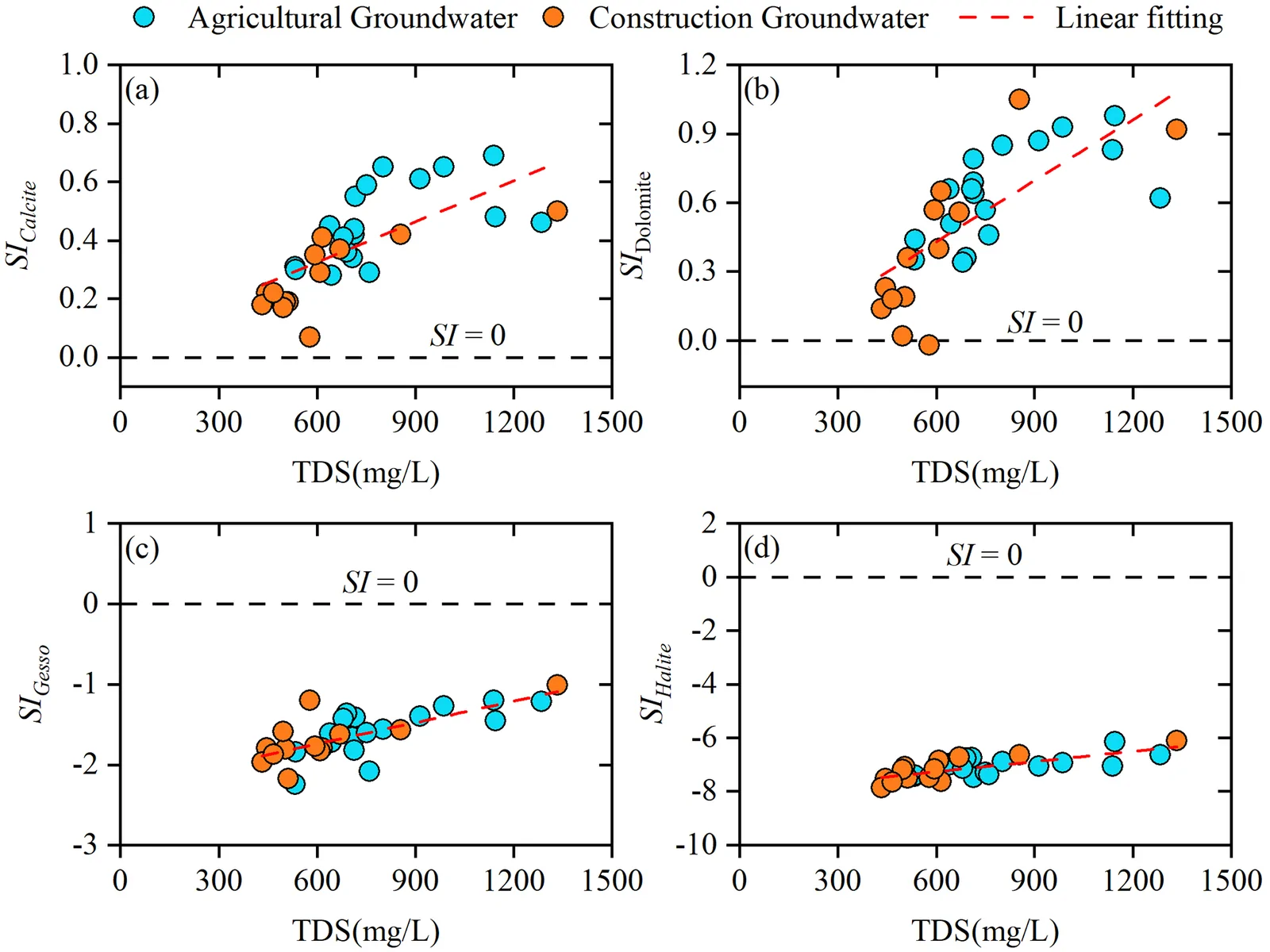
Relationship between mineral saturation index and TDS.

#### Cation exchange.

Cation exchange adsorption is a process in which the cations on the surface of rock are replaced by those in water during the interaction between water and rock, thus changing the chemical composition of water [38]. CAI serves as a key indicator for characterizing the type and intensity of cation exchange reactions at the water-rock interface, with its mathematical expression defined by Equations (2) and (3). From a hydrochemical kinetics perspective, when both CAI-I and CAI-II exhibit negative values, it signifies the occurrence of positive cation exchange, where Na⁺ in the aquifer medium desorbs into the water phase while Ca^2+^ in the aqueous phase is adsorbed by the solid medium. Conversely, if both indices are positive, it indicates that reverse adsorption dominates, manifested as reverse cation exchange, in which Na^+^ in the aqueous phase is exchanged for Ca^2+^ in the solid phase [28]. Fig 9a shows a significant linear positive correlation between the summed cation and summed anion concentrations (in meq·L-1) of groundwater from both agricultural and construction lands. The equation y = 1.125x + 0.466 (R^2^ = 0.927), indicates a near 1:1 charge balance, confirming the internal consistency of the dataset.

**Fig 9:**
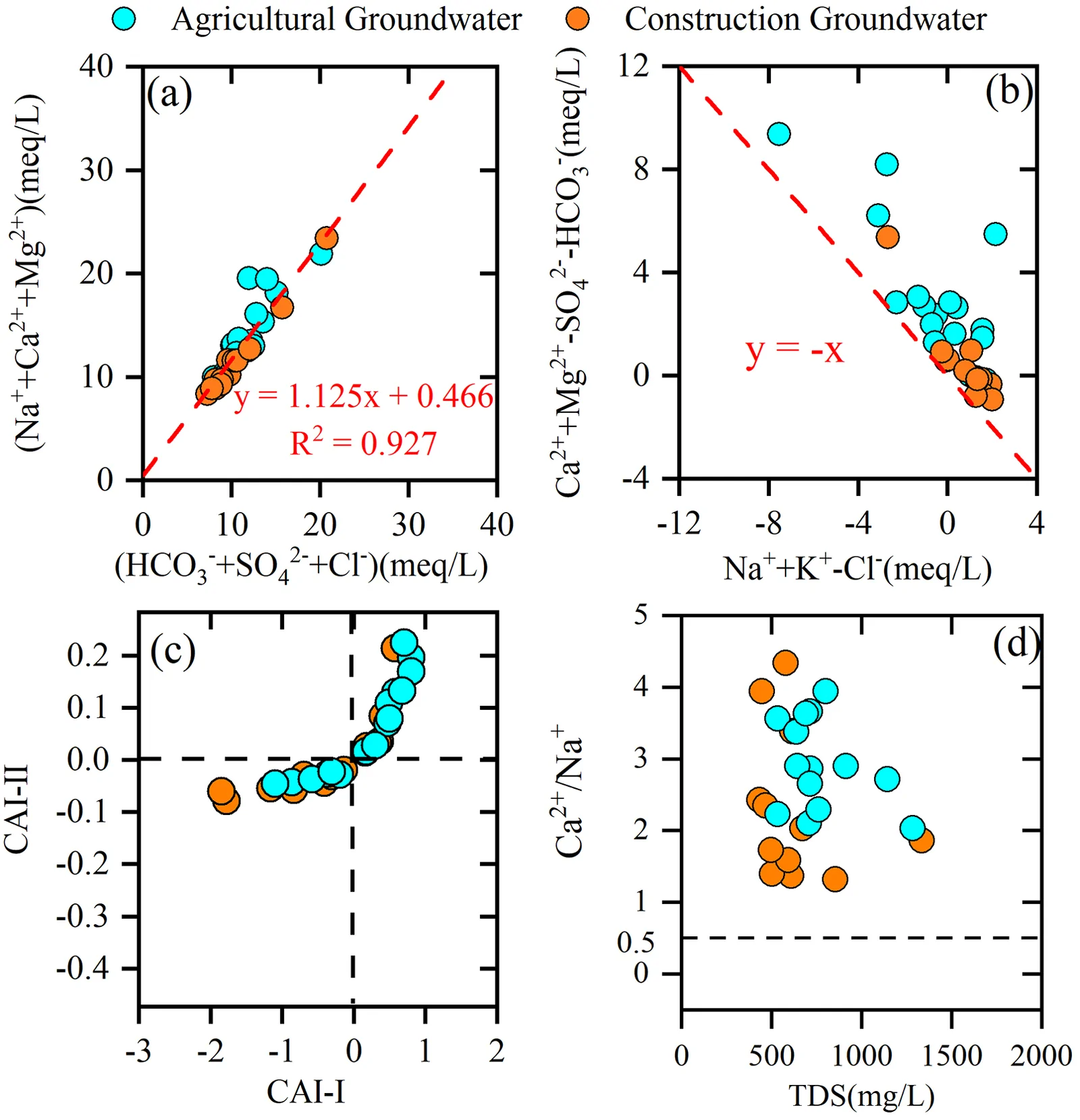
Ratio diagram of cation alternating adsorption.

Agricultural groundwater samples (Fig 9b) predominantly fall in the upper-left quadrant, exhibiting negative (Na^+^ + K^+^ − Cl^−^) values that are consistent with reverse cation exchange. This interpretation is further supported by the positive CAI values observed in most agricultural samples (Fig 9c) and Ca^2+^/Na^+^ ratios (Fig 9d), which collectively indicate Na⁺ adsorption onto the solid phase coupled with Ca^2+^ release into groundwater [28]. This reverse cation exchange is primarily driven by the elevated ionic strength of soil solutions under long-term irrigation and fertilization, which alters soil salinity saturation and promotes the replacement of adsorbed Ca^2+^ by Na ⁺ . In contrast, construction groundwater samples cluster in the lower-right region of Fig 9b, showing positive (Na^+^ + K^+^ − Cl^−^) values indicative of positive cation exchange. This pattern is corroborated by negative CAI values in Fig 9c and lower Ca^2+^/Na^+^ ratio in Fig 9d, reflecting Na^+^ desorption from the aquifer matrix into groundwater under conditions of lower ionic strength and shorter water-rock contact times typical of urbanized settings with impervious surfaces. The foregoing natural geochemical processes provide the hydrochemical baseline upon which anthropogenic disturbances are superimposed. The following section examines how agricultural and urban activities modify this baseline through specific ionic inputs.

### Human activity impacts

According to the aforementioned analysis, agricultural and construction activities appear to exert an important influence on groundwater chemistry. The sensitivity of groundwater chemistry to land-use changes is particularly pronounced in eco-sensitive regions where natural water-rock interactions are delicately balanced. Bakshe et al. demonstrated that even moderate anthropogenic disturbances in the Western Ghats markedly altered groundwater ion dynamics [3]. This is typically reflected in changes in the concentrations of Na^+^, SO_4_^2-^ and NO_3_^-^. Cl^-^ and NO_3_^-^ may be influenced by domestic sewage discharge and nitrogen fertilizers, whereas SO_4_^2-^ may reflect both background geogenic inputs and additional anthropogenic contributions [39]. By calculating the ratios of (SO_4_^2-^)/(Na^+^) and (NO_3_^-^)/(Na^+^), a higher ratio indicates a stronger influence of human activities on the water's chemical composition [40,41].

As shown in Fig 10a, the (NO_3_^-^)/(Na^+^) ratio in agricultural groundwater exceeds that in construction groundwater, indicating that agricultural activities, particularly the application of nitrogen fertilizers, substantially elevate nitrate levels in the soil and groundwater. This reflects the intensive use of nitrogen fertilizers in agricultural groundwater, which increases the concentration of NO_3_^-^ in the groundwater. In contrast, construction groundwater exhibits a lower (NO_3_^-^)/(Na^+^) ratio, suggesting that nitrate contamination is less pronounced here. The relatively lower NO_3_^-^ levels of construction groundwater are likely due to the absence of extensive agricultural inputs, with more influence from industrial pollution or sewage discharge, which may contribute other pollutants, including chloride and sulfate. In contrast, the (SO_4_^2-^)/(Na^+^) ratio shows minimal variation between agricultural land and construction land. This suggests that SO_4_^2-^ levels in both areas are similarly influenced by processes like industrial pollution (e.g., sulfur emissions from coal combustion) or natural sources such as the dissolution of gypsum or other sulfur-bearing minerals. However, elevated sulfate levels were observed at the QS05 sampling point located near Jiezhuang Street, which may reflect localized hydrogeochemical or anthropogenic influences.

**Fig 10:**
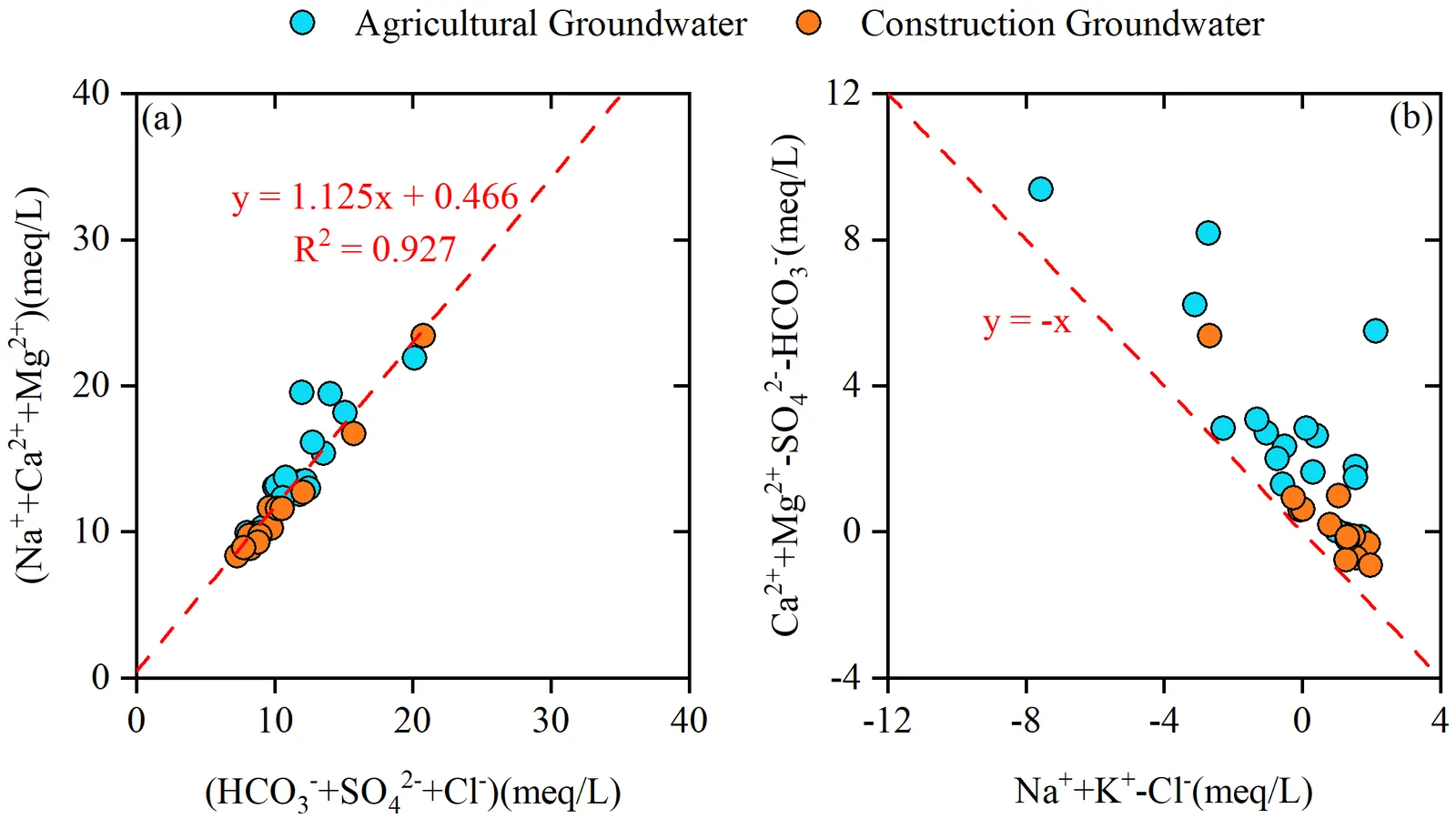
(SO_4_^2-^)/(Na^+^) versus (NO_3_^-^)/(Na^+^) and (NO_3_^-^)/(Na^+^) versus Cl^-^.

Based on the previous analysis, human activities are the primary drivers of changes in the chemical composition of groundwater. To further differentiate the impacts of industrial activities, agricultural activities, and domestic sewage on groundwater chemistry, the ratio of (NO_3_^-^)/(Na^+^) to Cl^-^ can be used for identification [42]. Fig 10b reveals that in the study area, the (NO_3_^-^)/(Na^+^) ratio for agricultural groundwater is relatively high, with all data points showing a ratio above 1 in contiguous agricultural land areas. This strongly suggests that agricultural activities are an important source of nitrate enrichment in these areas. In contrast, a low ratio in construction groundwater may reflect mixed influences, including domestic sewage, urban-industrial inputs, or possible biogeochemical attenuation such as denitrification.

In synthesis, agricultural groundwater is governed by carbonate dissolution amplified by irrigation return flow, yielding higher Ca^2+^, Mg^2+^, HCO_3_^-^ and nitrate enrichment under reverse cation exchange; construction groundwater, by contrast, shifts toward silicate-weathering and Na^+^-Cl^-^-coupled signatures under positive cation exchange and shorter residence times typical of impervious urban surfaces. These divergent trajectories reflect differing human forcing intensities rather than a single monotonic trend.

### Limitations of this study

First, the study relies on single-time sampling (February–March 2021), which precludes assessment of seasonal or inter-annual hydrochemical dynamics. Groundwater quality in this region may respond strongly to seasonal precipitation patterns, fertilizer application cycles, and extreme climate events, all of which could introduce temporal bias in the observed land-use-related differences. Second, while ion ratio analysis effectively identifies dominant control processes, it cannot precisely apportion contributions from multiple overlapping sources in “mixed-zone” samples, nor can it distinguish between physical dilution and biogeochemical attenuation (e.g., denitrification) on the basis of concentration data alone. Third, the limited sample size (n = 31) constrains the statistical power for detecting subtle hydrochemical differences and may not fully capture localized heterogeneity within each land-use category.

Despite these limitations, this study contributes an integrated analytical framework that combines land-use data with multiple geochemical mapping methods to rapidly identify regional anthropogenic disturbance intensity. In the absence of systematic baseline water quality data in the Jining High-tech Zone, the spatial distribution patterns and source apportionment results presented here provide an essential scientific foundation for preliminary groundwater resource protection policies. Future research should establish long-term monitoring networks spanning multi-year hydrological cycles and incorporate multi-isotope tracing combined with microbial community analysis to quantify source contribution ratios and biogeochemical reaction rates more precisely.

## Conclusions

This study demonstrates that shallow groundwater in the Jining High-tech Zone, a rapidly urbanizing region where agriculture, industry, and built-up areas coexist, exhibits clear hydrochemical differences between agricultural land and construction land. Agricultural groundwater is characterized by higher concentrations of Ca^2+^, Mg^2+^, and HCO_3_^-^, reflecting the dominance of carbonate dissolution enhanced by agricultural irrigation return flow and fertilizer inputs. In contrast, construction groundwater displays greater spatial variability in TDS and a shift toward higher Na^+^ and mixed-anion facies, indicating stronger influences from silicate weathering, cation exchange, and localized anthropogenic disturbances including sewage leakage and urban infrastructure losses. Along the northeast-to-southwest groundwater flow path, ion concentrations progressively increase from recharge areas (HCO_3_-Ca·Mg type) to near the lakes (mixed HCO_3_·Cl-Ca·Mg and HCO_3_-Ca·Na types), demonstrating that hydrochemical evolution is jointly controlled by land-use practices and natural water-rock interactions along extended flow paths.

The principal contribution of this study lies in demonstrating that land-use type serves as a distinguishable but not exclusive control on shallow groundwater hydrochemistry: PCA revealed partial separation of agricultural and construction samples along the mineralization gradient (PC1), while substantial overlap along PC2 and PC3 confirms that natural hydrogeological factors including aquifer lithology, groundwater flow path length, and residence time exert equally important controls. This finding provides new insight into the coupled natural-anthropogenic framework governing land-use-related hydrochemical differentiation in shallow alluvial aquifer systems.

These findings have practical implications for groundwater protection and management in the Jining High-tech Zone. For agricultural land, priority should be given to controlling nitrate loading, given that NO_3_-N concentrations in agricultural groundwater reached a maximum of 108 mg/L, indicating substantial fertilizer-derived contamination. Precision nutrient management and vegetative buffer strips along the Sihe River and its tributaries would help intercept nitrogen-rich runoff before it recharges the shallow aquifer. For construction land, the prominent NaCl association identified through correlation and cluster analysis points to leakage from municipal sewage networks as a primary concern. Systematic infrastructure rehabilitation and stricter oversight of industrial wastewater discharge are needed to mitigate chloride and sulfate inputs. The divergent hydrochemical signatures identified in this study—carbonate-dominated with nitrate enrichment under agriculture versus silicate-influenced with sodium-chloride signatures under urbanization—provide measurable geochemical indicators for land-use-specific groundwater monitoring programs.

Future studies should incorporate multi-season sampling to assess temporal hydrochemical dynamics, combined with stable isotope tracing to elucidate biogeochemical reaction rates, thereby refining the process-based understanding established in this work.

## Supporting information

S1 Data Descriptive statistics and the underlying hydrochemical dataset. (XLSX)

S2 Data PCA summary comparison. PCA summary comparison of the pooled, agricultural and construction datasets.
(XLSX)
